# The effectiveness of information and communication technology-based psychological interventions for paediatric chronic pain: protocol for a systematic review, meta-analysis and intervention content analysis

**DOI:** 10.1186/s13643-016-0350-1

**Published:** 2016-10-18

**Authors:** Angeline Traynor, Eimear Morrissey, Jonathan Egan, Brian E. McGuire

**Affiliations:** 1 School of Psychology, National University of Ireland, Galway, Ireland; 2 Centre for Pain Research, National University of Ireland, Galway, Ireland

**Keywords:** Cognitive behavioural therapy, Psychological, Chronic pain, Meta-analysis, Systematic review, Randomised controlled trials, Internet, Online, Information technology

## Abstract

**Background:**

Resource and geographic barriers are the commonly cited constraints preventing the uptake of psychological treatment for chronic pain management. For adults, there is some evidence to support the use of information and communication technology (ICT) as a mode of treatment delivery. However, mixed findings have been reported for the effectiveness and acceptability of psychological interventions delivered using information and communication technology for children and adolescents. This is a protocol for a review that aims to (i) evaluate the effectiveness of psychological interventions delivered using information and communication technology for children and adolescents with chronic pain and (ii) identify the intervention components and usability factors in technology-based treatments associated with behaviour change.

**Methods/design:**

We will conduct a systematic review to evaluate the effectiveness of psychological interventions for paediatric chronic pain delivered using ICT. We plan to directly compare ICT-based, psychological interventions with active control, treatment as usual or waiting list control conditions. This systematic review will be reported in line with the Preferred Reporting Items for Systematic Reviews and Meta-Analyses (PRISMA) guidance. Published and unpublished randomised controlled trials will be included and the literature search will comprise Ovid MEDLINE, Ovid Embase, PsycINFO and the Cochrane Library on Wiley, including CENTRAL and Cochrane Database of Systematic Reviews. Grey literature including theses, dissertations, technical and research reports will also be examined. Two review authors will independently conduct study selection, relevant data extraction and assessment of methodological quality. Risk of bias in included studies will be assessed using the Cochrane Collaboration risk of bias tool criteria. Two qualified coders will independently code behaviour change techniques according to the behaviour change taxonomy (v1) of 93 hierarchically clustered techniques and a novel coding scheme for mode of delivery and usability factors. A quantitative synthesis will be conducted if appropriate.

**Discussion:**

The findings of this review may offer insight for healthcare professionals working in chronic pain services and to researchers involved in designing and evaluating information and communication technology-based interventions.

**Systematic review registration:**

PROSPERO CRD42016017657

**Electronic supplementary material:**

The online version of this article (doi:10.1186/s13643-016-0350-1) contains supplementary material, which is available to authorized users.

## Background

### Description of the condition

Chronic pain is defined by the International Association for the Study of Pain (IASP) as ‘an unpleasant sensory and emotional experience associated with actual or potential tissue damage, or described in terms of such damage’ [1]. In practice and across the literature, chronic pain is widely accepted to be pain which persists for a period of 3 months or more. Among children and adolescents, recent reports suggest it continues to be highly prevalent. Current prevalence rates estimate between 11 and 38 % of children and young people are affected by chronic or persistent pain [2–4]. Pain in childhood is thought to be a major public health concern across Western countries, particularly because prevalence has been found to increase with age and is predictive of persistent pain in adulthood [2–4]. The most commonly reported paediatric pain conditions include headache, abdominal pain, musculoskeletal pain and multiple or widespread pain. Across pain types, prevalence is generally higher in girls and those with lower socioeconomic status [2–4].

Chronic pain has an enormous impact on the lives of children and on family functioning in general. Approximately 5–15 % of those affected by paediatric chronic pain report severe and debilitating levels of pain [5, 6]. High pain levels are associated with extensive and often sustained negative effects on child health and overall quality of life for the child and family. Severe chronic pain interferes with daily functioning [7], sleep [8, 9], emotion regulation [10], social functioning [11], school performance and attendance [12] and family functioning [13–16]. Psychological therapies have been found to be effective in reducing pain and disability in young people with chronic pain [17, 18]. However, many children and young people do not have access to psychological services to support pain management. Commonly reported barriers include a lack of access to trained healthcare professionals, financial constraints, geographic barriers and scheduling issues. There is some evidence to suggest technology-facilitated delivery of psychological interventions may help resolve some of the current health care access issues [19].

The reach of technology-delivered treatment could be extensive. A recent Quarterly National Household Survey (QNHS; 2015) of internet usage in the home found that 85 % of households in Ireland have access to the internet [20]. Estimates for Europe and North America range from 74 to 88 %, and worldwide, this figure is approximately 46 % [21]. The advantages of online delivery of interventions include increased convenience for users, reduction of health service costs and isolation of users, the provision of timely information, increased user and supplier control of the intervention and research-related benefits [19]. However, little is known about the impact of change in mode of delivery from traditional face to face to technology-based treatment. As pointed out by Keogh, Rosser and Eccleston (2010), there is little guidance on how to translate therapy from traditional, human-mediated delivery to technology-based platforms [22].

### Description of the intervention

Psychological treatment for chronic pain typically involves a combination of evidence-based cognitive and behavioural strategies such as relaxation training, cognitive restructuring, acceptance-based skills, information and social support. Some techniques, for example, cognitive restructuring, are likely to improve symptoms by influencing how the individual interprets and attributes meaning and emotion to the sensation of pain. Other strategies aim to reduce muscle tension and physiological arousal and thereby promote more adaptive response to pain symptoms.

Although the number of information and communication technology (ICT)-based interventions for paediatric populations is increasing [23–28], as a mode of treatment delivery for pain management, technology-based methods are still very much in their infancy. Research indicates children and adolescents may be particularly amenable to technology-delivered treatment given their reported comfort with, and time spent using, digital technologies [29]. Evaluations of existing examples are mixed. Many ICT interventions focus on adult pain populations [30], some demonstrate promising findings [31] and others are exploratory or have yet to be extensively evaluated for their effectiveness [32–34]. Qualitative reports and treatment satisfaction data would seem to support this conclusion, suggesting technology-based treatment delivery is acceptable to young people with chronic conditions [35, 36], However, high attrition and low adherence rates are reported across the literature and suggest further evaluation of the importance of contact with therapist and the acceptability of online or technology-based delivery of treatment is necessary [25, 28, 37–39]. Other than the recent review by Fisher et al. (2015), few studies have evaluated ICT-delivered therapies for the management of paediatric chronic pain compared to traditional face to face therapies. In addition to larger sample sizes and the inclusion of active comparators, Fisher and colleagues call for further investigation that allows a better understanding of effective features of ICT-based interventions [38].

### How the intervention might work

Treatment effects may result from the provision of evidence-based psychological treatment and may also be facilitated by the characteristics of treatment delivery. The effectiveness of ICT-delivered treatment may be related to the presence and combination of specific behaviour change techniques and how they are implemented or the usability of the platform in terms of efficiency, learnability, satisfaction or personalisation. Health benefits in the form of behaviour change or symptom improvement associated with ICT-interventions may be attributable to an amalgamation of user characteristics, environmental factors and the presence of human support in the form or an e-coach or online therapist [40, 41]. It may also be that the use of engaging and interactive technology in treatment encourages children to perceive therapeutic instruction in terms of discovery. The personification of an e-coach or online therapist in the form of an avatar or friendly image may encourage the perception of the therapist as a facilitator rather than a teacher or figure of authority. Health benefits may also be boosted by the lack of time constraints or unlimited access typically associated with online interventions. The freedom to access the intervention at a convenient time may facilitate the delivery of timely information and promote the therapeutic learning or practice as an ongoing or long-term habit rather than a scheduled classroom- or clinic-based activity [29, 42].

### Why it is important to do this review

Chronic pain may present as a result of injury, infection or surgical procedure, and often, no apparent cause is found. Despite the personal and economic burden of paediatric chronic pain for the individual and family as a whole, it is often seen as symptom rather than a condition in its own right [43]. Perhaps as a consequence, chronic pain and particularly paediatric chronic pain does not receive the same focus or priority given to other chronic conditions. Efforts are needed to determine how interventions should be developed to address paediatric chronic pain management. It will be important to determine whether appropriate, effective ICT-based pain management interventions can be identified, in order that effective therapies can be developed and distributed to improve pain symptoms in school age children. In the context of a rapidly expanding suite of apps and other ICT-based clinical tools, we believe it is important to determine the acceptability and effectiveness of ICT-delivered therapies compared with traditional face to face therapies. Identifying the intervention technology components and behaviour change techniques used in current interventions will contribute to the evidence base for the development of with ICT-based, paediatric pain management interventions.

### Purpose of the proposed review

The purpose of the proposed systematic review is to evaluate the effectiveness of psychological interventions for paediatric chronic pain delivered using ICT. We plan to directly compare ICT-delivered interventions with active control, treatment as usual or waiting list control conditions. We will assess treatment efficacy based on the PedIMMPACT recommendations [44] and the recommended IMMPACT criteria outlined for interpreting the clinical importance of treatment outcomes in chronic pain clinical trials [45]. Our primary outcome of interest will be pain interference (i.e., reduced disability) and pain intensity. Our secondary outcomes will be emotional functioning, global rating of improvement, quality of life, adverse events and treatment satisfaction. We hypothesise that ICT-delivered interventions will differ in terms of treatment benefits for both our primary and secondary outcome measures of interests. This review protocol parallels that of Eccleston, Fisher, Craig, Duggan, Rosser and Keogh [30, 46] which focuses on telemedicine for chronic pain management in adults. It also builds on the recently published systematic review by Fisher et al. (2015) which focuses on the remote delivery of psychological therapies for paediatric chronic pain in children and adolescents. The proposed review will go beyond previous evidence syntheses by exploring putative intervention components and usability factors which may act as potential sources of variation in effects.

### Aim

This systematic review will evaluate the features and effectiveness of psychological interventions for paediatric chronic pain delivered using ICT.

## Key objectives

The key objectives of this study are the following:

1. To evaluate the effectiveness of psychological interventions for paediatric chronic pain delivered using ICT in comparison with active control, treatment as usual or waiting list control conditions
2. To identify the intervention components (theoretical basis, behaviour change techniques, interactive elements and level of human support) associated with effectiveness in ICT-based interventions relative to active control, treatment as usual or waiting list control conditions.

## Methods

### Criteria for considering studies for this review

The conduct and report of the proposed review and meta-analysis will adhere to the reporting guidelines of the “Preferred Reporting Items for Systematic Reviews and Meta-analyses Protocols (PRISMA-P) 2015 Statement” and the PRISMA statement (see Additional file 1) [47, 48]. This systematic review protocol was registered with the International Prospective Register of Systematic Reviews (PROSPERO) database (CRD42016017657).

### Types of studies

We will assess all published and unpublished randomised controlled trials (RCTs) which evaluate the effectiveness of ICT-based psychological interventions for paediatric chronic pain management. This will include treatment groups compared with active control, treatment as usual or waiting list control.

### Type of participants

We will focus on children and adolescents 18 years of age and younger that meet the criteria for a diagnosis of non-malignant chronic pain. In the absence of a clinical diagnosis, chronic pain will be defined as self-reported persistent pain lasting 3 months or more. This is consistent with the definition of chronic pain provided by the International Association for the Study of Pain, which states: pain is an ‘unpleasant sensory and emotional experience, associated with actual or potential tissue damage, or described in terms of such damage’ [1]. Chronic pain conditions may include headache or migraine, pain in any body area and pain associated with a range of conditions (e.g. rheumatoid arthritis, myofascial pain conditions, neuralgia, fibromyalgia). Studies that enrolled adults or patients who had been experiencing pain for less than the 3-month threshold duration will be excluded from the present review.

### Type of interventions

We will include interventions which evaluate the effect of psychological treatment for chronic pain in children and adolescents, delivered using ICT. At least one arm of each included trial must involve a predominantly psychological therapy or include definable psychotherapeutic content. In line with McGuire et al. (2014), psychological interventions will be included if they use techniques often used for chronic pain management including relaxation training, cognitive restructuring (i.e. changing pain-related beliefs, reducing catastrophic thinking), setting and working towards behavioural goals (e.g., exercise), behavioural activation and problem-solving. Studies that use methods such as meditation, mindfulness, stress management or other techniques to improve pain self-management will be included. All interventions must aim to reduce pain characteristics, functional limitations, psychological distress and/or more adaptive behaviour change. A measure of pain characteristics must be included in any examination of the effects of psychological treatment on multiple outcomes (see the ‘Type of outcomes measures’ section below).

### Types of intervention delivery

Studies must evaluate ICT-based interventions which function as the primary mode of treatment delivery. Interventions which involve the support of a health care professional will only be considered if the primary mode of treatment delivery is ICT-based. Studies where ICT is used to facilitate traditional treatment but does function as the primary source of treatment (e.g. aid symptom monitoring or communication only) will be excluded. No restriction will be placed on the level of user interaction or data input in a given ICT-based, intervention platform. We will include studies that evaluate any information and communication-based intervention delivered in the home, school or community.

### Type of outcome measures

#### Primary outcomes

The primary outcomes of interest are those that measure change in pain characteristics including intensity, severity or frequency and pain interference including physical and social interference (e.g. related school absenteeism) from pre to post treatment. Pain intensity, severity and frequency are generally measured using self-reported verbal or numerical rating scales or visual analogue scales. Pain interference is commonly measured using psychometric tools with established validity and reliability, e.g. Functional Disability Inventory (FDI) [49] or the Pediatric Quality of Life Inventory (PedsQL) [50]. In cases where the chronic pain condition refers to chronic headache pain or migraine, psychometric tools such as the PedMIDAS [51] may be used. As per Eccleston et al. (2014) and McGuire et al. (2014), we will also report the responder rate (the percentage of subjects in the treatment group with at least 50 % reduction in the primary efficacy measure) [30, 52].

#### Secondary outcomes

Secondary outcomes of interest include emotional functioning (self-reported measures of psychological distress such as depression and/or anxiety), quality of life (self-report questionnaires assessing the impact of chronic pain on quality of life) and global ratings of improvement (self-reported measure of change in subjective sense of wellbeing). Data relating to treatment acceptability and satisfaction, retention and attrition will also be extracted as a secondary outcome. Adverse events will also be reported. These variables may be measured using psychometric tools with established validity and reliability such as the Paediatric Quality of Life Inventory (PedsQL) [50], the Children’s Depression Inventory (CDI) [53]. Finally, we will include variables which are measured as a discrete outcome or as a subscale within a composite measure.

### Search methods for identification of studies

No restrictions will be placed on the date of publication or publication status. Studies will be included if the full report is accessible in English, either through electronic search or through contact with the author.

We will design and conduct a three-step search strategy using methods recommended by the Cochrane Collaboration [54].

1. The initial search strategy will be designed with consideration of other similar reviews’ strategies. In addition, we will conduct an initial search of journals indexed in MEDLINE and PsycINFO with the aim of extracting and compiling a comprehensive list of text or key words contained in the title, abstract and subject descriptors/MeSH terms of relevant articles. All identified key words and their synonyms will be compiled and used to develop an individual search strategy for MEDLINE. This will be revised appropriately for each database searched (see Table 1).

**Table 1:** Details of search strategy

Search item
1. (child or adolescent or infant or juvenile or pediatric or paediatric or young person or young people or youth or young adult or teen or teenager or boy or girl or schoolchild)
2. (psychology or psychotherapy or behavior therapy or cognitive or hypnosis or relaxation or mindfulness or meditation or acceptance or imagery)
3. (chronic pain or recurrent pain or persistent pain or nociceptive pain or psychogenic pain or neuropathic pain or somatic pain or pain or headache or migraine or cephalagi or neuralgi or arthritis or juvenile idiopathic arthritis or JIA or juvenile fibromyalgia syndrome or fibromyalgia or ankylosing spondylitis or juvenile spondylitis or rheumatoid arthritis or osteomyelitis or chronic pelvic pain or non-cardiac chest pain or complex regional pain syndrome or mixed pain or neuropathic pain or mixed pain or musculoskeletal pain or knee pain or back pain or low back pain or stomach ache or tummy ache or abdominal and pain or belly ache or recurrent abdominal pain or ear ache or odontalgi or myofascial and pain or orofacial pain or facial pain)
4. (Internet or Telecommunications or telemedicine or telemedicine or telehealth or tele-health or e-health or ehealth or world and wide and web or www or web-based or email or e-mail or online or social media or computer or technology software or telephone or smartphone or cellphone or mobile or mobile health or mhealth or m-health or text or app or ICT or information and communication technology or interactive or virtual reality or VR or augmented reality or AR or game based or gamification)
2. We will search several databases including OVID MEDLINE®, OVID EMBASE, OVID PsycINFO and the Cochrane Central Register of Controlled Trials (CENTRAL). The databases will be searched for randomised controlled trials of ICT-delivered interventions for paediatric chronic pain conditions through the time period of database inception to the present.
  Clinical trial registries will be searched to identify completed and in-progress trials. This will include the following databases:
  ClinicalTrials.gov (clinicaltrials.gov)
  The metaRegister of controlled trials (mRCT), (controlled-trials.com)
  The World Health Organization (WHO) International Clinical Trials Registry Platform (ICTRP) (www.who.int/ictrp/en/) for trials.
  Grey literature including theses, dissertations, research and technical reports and conference papers will also be examined. Grey literature source selection will be guided by CADTH’s “Grey matters: a practical search tool for evidence-based medicine” [55]. This will include the following databases:
  Scopus (https://www.scopus.com)
  ProQuest (www.proquest.com)
  Ethos (http://ethos.bl.uk)
  Open Grey (http://www.opengrey.eu)
  TRIP (Turning Research into Practice http://www.tripdatabase.com);
  WorldCat (www.worldcat.org)
  National Technical Information Service (NTIS, http://www.ntis.gov/)
3. The initial electronic search strategy will be supplemented by screening the reference lists of included reports and articles to identify additional studies. If not established through other methods, authors will be contacted for details regarding the status of a given study.

### Data collection and analysis

#### Selection of studies

The titles and abstracts of publications obtained by the search strategy will be independently screened by two authors (AT, BMG). Those that fail to meet the outlined inclusion criteria will be removed. All remaining publications will be retrieved for further scrutiny. Two review authors (AT, BMG) will independently assess the full text of studies which initially meet the review criteria. Disagreements between review authors will be discussed until resolved; in the event a resolution cannot be reached, a third review author will arbitrate (JE). A record will be kept of all articles excluded at this stage and the reason for their exclusion. We will produce a PRISMA flow diagram to illustrate the search and systematic review process as recommended in Chapter 6 of the Cochrane Handbook [54].

#### Data extraction and management

A data extraction form will be created prior to data extraction. Data will be extracted independently by one reviewer (AT) and verified by another (BMG) using a customised form, which will be piloted prior to use. Disagreements in data extraction will be resolved through discussion with the primary data extractor, as required. In the event that resolution cannot be reached, a third review author will arbitrate (JE). The finalised data will be entered into RevMan 5.3 [56]. Multiple publications of the same study will be identified, linked and used for all relevant reported data. In such cases, the original publication will be given priority. Where the necessary outcome data are unavailable, we will contact study authors. If the data remains unavailable, the study will not be included in any assessment. The authors will not be blind to the study author, institution or journal.

We will extract data relevant to the following categories: (i) Study population and design, (ii) Intervention and (iii) Outcome. Characteristics of included studies’ table(s) will be created and may include the following information:

- Participant characteristics
- Geographic location
- Assessment periods
- Description of providers of intervention and comparison interventions
- Primary and secondary outcomes
- Theoretical basis (domains identified)
- Therapeutic content (characteristics of psychological therapies)
- Mode of delivery (Internet, smartphone app, telephone, text)
- Behaviour change techniques
- Control condition
- Intensity (e.g. no. of sessions, total contact time, duration)
- Treatment engagement (retention and attrition)

The use of theory in the included interventions will be coded according to the Theoretical Domains Framework (TDF) [57]. This is an integrative framework which was developed and validated by Cane, O’Connor and Michie (2012). The TDF summarises the range of psychological theory potentially driving behaviour change, into a total of 14 distinct domains. In line with the approach taken by Little et al. (2015), descriptions of intervention and control conditions will be assessed to determine if and to what extent TDF domains are targeted within [58]. This process will be carried out independently by two coders (AT, EM) using a data extraction form similar to the form used by Little et al. (2015). Inter-rater reliability will be calculated and discrepancies will be discussed until resolved.

The behaviour change technique taxonomy (v1) of 93 hierarchical clustered techniques [59] will be used to code intervention content. Mode of delivery and usability factors will be coded using a novel coding scheme adapted by Webb et al. (2010) and van Genugten (2016) [60, 61]. Two qualified coders (AT, EM) will independently code the behaviour change techniques described in the intervention and control conditions. Kappa and percentage disagreement will be calculated. Disagreements between reviewers will be discussed until resolved or with third party arbitration (BMG) if required.

#### Assessment of risk of bias

For each included study the review authors (AT, BMG) will independently carry out a domain-specific assessment of risk of bias using the recommended Cochrane Collaboration’s tool for risk of bias assessment [54]. This will involve the classification of risk of bias in included studies as ‘low’, ‘unclear’ or ‘high’ risk of bias. If the authors (AT, BMG) disagree the final rating will be made by consensus with the third author (JE). The domains assessed will include:

- Random sequence generation—to assess the potential for selection bias
- Random allocation concealment—to assess the potential for selection bias
- Blinding of participants and personnel—to assess the potential for performance bias (both participants and outcome assessors)
- Blinding of outcome assessment—to assess the potential for detection bias
- Incomplete outcome data—to assess the potential for attrition bias
- Selective reporting—to assess the potential for reporting bias
- Other bias—to assess the potential for other sources of bias not covered in other domains

Where necessary, we will contact the study authors to request missing data and/or data clarification. The quality of the data included in the review and the presence of any serious flaws will be reported.

#### Overall quality of the evidence

If appropriate, we will use the Grading of Recommendations Assessment, Development and Evaluation (GRADE) approach to summarise the quality of evidence for each outcome at post-treatment and follow-up [54, 62, 63]. We will use GRADEprofiler (GRADEpro) to import data from Rev Man 5.3 to create Summary of Findings table(s) [54, 64, 65]. This will report outcome-specific information concerning the overall quality of evidence (risk of bias, inconsistency, imprecision and indirectness). Only the most important outcomes will be included in each ‘Summary of findings’ table. As per Fisher et al. (2015), the most important outcomes will be deemed those with the largest number of participants in each arm [38].

#### Sensitivity analysis

Sensitivity analysis based on methodological rigour and risk of bias will be carried out to determine the robustness of results. Studies deemed to be of high or unclear risk of bias across different domains will be systematically excluded then included, in order to assess differences in the overall effect estimates. If no significant differences exist, the studies will be included in the main analysis.

#### Measures of treatment effect

In order to synthesise data across studies, we will compute and report mean differences where identical scales are used to measure the same clinical outcome. Where different scales are used to measure the same clinical outcome, we will compute standardised mean differences (SMDs) (otherwise weighted mean differences). For dichotomous data, we will report odds ratios (ORs), 95 % confidence intervals (CIs) and number needed to treat to benefit (NNTB). For continuous data we will most likely report standardised mean differences and 95 % confidence intervals. There are a small number of studies in this area. We expect data to be sparse, event rates may be low or study size may be small therefore we will use Mantel-Haenszel methods in analyses of dichotomous data. We anticipate effect sizes will be similar but not identical across studies therefore a random-effects model will be used in analyses. Pain-related interference and pain intensity outcomes tend to be reported using primarily continuous data and studies which include headache conditions are likely to report pain symptoms using primarily dichotomous data. Chronic pain conditions will be categorised according to pain condition. In line with Fisher et al. (2015), data from studies reporting mixed pain conditions will be entered into all appropriate analyses. When studies use more than one measure for a given outcome, we will extract the most reliable or widely accepted. As per Eccleston et al. (2014) and Fisher et al. (2015), the timeframe allowed for collection of follow-up data will range from 3 to 12 months’ post treatment. If more than one-time point is available at follow-up, the latest data collection point will be extracted.

#### Missing data

Where necessary, attempts will be made to contact the lead authors of included studies to request missing data. We will, where necessary, calculate standard deviations using the methods described in the Cochrane Handbook for Systematic Reviews of Interventions (2011). Included studies will be scanned for other statistics including confidence intervals, standard errors or *p* values that would allow for its calculation. If missing data required for analyses cannot be obtained from the study author or extrapolated from other statistics, the study will be excluded. We will record the use of intention to treat analyses (ITT) and, if sufficient data is available, we will conduct subgroup analyses to evaluate the impact of inclusion or exclusion of non-completers in final study analysis.

We will report rates of missing outcome data per arm and refer to the Cochrane risk of bias tool for missing outcome data in any evaluation of imputation methods. Finally, we address the potential impact of missing data on the findings of the review in the ‘Discussion’ section [54].

#### Unit of analysis issues

We anticipate unit of analysis issues such as repeated observations of the same outcome and studies including multiple intervention arms. For studies reporting repeated measurements of the same outcome, we will extract data at the following time points: baseline, post-treatment (not longer than 3 months post-randomisation) and follow-up (not longer than 12 months post-randomisation). For studies including more than two intervention groups, we will adhere to the recommended method suggested by the Cochrane Collaboration in section 16.5 for combining multiple groups from one study [54]. If cluster-randomised controlled trials are included, we will check for unit of analysis errors. Where possible, we will recalculate results using the appropriate unit of analysis (Higgins 2011). As per the Eccleston et al., (2012) protocol, all psychological intervention conditions will be labelled ‘treatment’ and all comparator conditions will be labelled ‘control’ conditions.

#### Assessing for heterogeneity

We will assess heterogeneity by calculating *χ* ^2^ and *I* ^2^ values for all outcome variables. Statistical heterogeneity will be considered substantial if *I* ^2^ values are above 50 %. We will also assess the impact of heterogeneity through sensitivity analyses and assume the appropriate random-effects or fixed-effect model in meta-analyses accordingly [54].

#### Assessment of reporting biases

According to section 10.1 of the Cochrane Handbook, reporting biases arise when dissemination of findings is influenced by the nature and direction of results [54]. For this reason, we will, where possible, retrieve and compare the protocol for the included studies with the final reports.

The potential for small study effects such as publication bias will be assessed visually by inspection of funnel plots of estimated effects by standard error and using statistical tests which are in line with recent recommendations [66, 67]. Funnel plots will be assessed if ten or more studies are identified. The possible reasons for asymmetry will be investigated.

#### Data synthesis

We will pool data using the Cochrane Collaboration’s Review Manager Software, RevMan 5.3 (RevMan 2014). A quantitative synthesis will be carried out only if the included studies are sufficiently homogenous in terms of quality, study design, participants, interventions, outcomes and type of analyses to provide a meaningful summary of effects. A narrative synthesis will be carried out if there is insufficient data to justify a formal meta-analysis. For continuous data, we will calculate and report standardised mean differences (SMDs) and 95 % confidence intervals (CIs). For dichotomous data, we will calculate and report odds ratios (ORs), 95 % confidence intervals (CIs) and number needed to treat to benefit (NNTB). In the event that data is sparse, we will use Mantel-Haenszel methods in analyses of dichotomous outcomes. Given the likely differences in interventions, comparators and participants we expect sufficient clinical heterogeneity that included studies will estimate different but related intervention effects. As some heterogeneity is inevitable we anticipate a random-effects model will be used in analyses.

In line with Little et al. (2015), a Pearson correlation (two-tailed) will be used to explore the relationship between the total number and frequency of different TDF domains coded and the effect size of the intervention for both the ICT-based interventions and the control conditions. For example, the number of different domains coded in the control group will be subtracted from the number of different domains coded in the intervention group. Sensitivity analysis will be used to investigate whether subtracting domains that appear in the control group impacts on the findings [58]. If there are no significant differences, data synthesis will be descriptive e.g. the proportion of studies that target specific domains will be identified.

#### Subgroup analysis and investigation of heterogeneity

If sufficient data are available, several subgroup analyses will be performed. The following factors will be examined in subgroup analyses to determine their effect on the response to a psychological intervention for paediatric chronic pain:

- Technology type - differences between modes of delivery including text messaging, online websites, virtual or game-based programmes, smartphone applications and telephone-based treatment delivery methods.
- Contact with therapist—the degree of guidance which features in each intervention, ranging from ‘pure’ and unsupported to ‘guided’ and frequently supported self-management interventions.
- Pain type—differences between pain type including arthritis, back pain, abdominal pain, mixed pain and headache pain
- Behaviour change techniques used (based on the findings of previous studies which suggest specific techniques are associated with effectiveness)
- Usability factors used (based on the findings of previous studies which suggest specific factors are associated with effectiveness)

Of these subgroup analyses, modes of delivery and the extent of personalised contact with the therapist may be the most important because it remains unknown which ICT-based intervention types are most effective for paediatric chronic pain management and also how effective personalised contact with therapist (e.g. an e-coach) is in comparison with pure (e.g. no contact with therapist) self-led programmes.

## Discussion

ICT-based psychological interventions may have the potential to address both the pain and disability associated with chronic pain conditions and the resource and geographic barriers to uptake of psychological treatment for chronic pain management. Reviews which identify effective components of ICT-based interventions have tended to focus on physical activity [68] or multiple behaviours [60], but to date these have not focused on chronic pain populations . This review will be the first to our knowledge, to evaluate the components, usability and effectiveness of ICT-based psychological interventions for children and adolescents with chronic pain. The findings of this review will offer insight for those involved in the design and development of complex psychological and technology-based interventions.

## Limitations

The findings from the current study will have certain limitations. First, we anticipate a small number of studies will be included. Second, it is expected that some interventions will fail to provide a detailed description of intervention content or to report explicit use of theory. This is a limitation of retrospective content coding. To address this issue, the lead authors of the included papers will be contacted and asked to provide more information. Also, content analyses will be conducted independently by two reviewers in an effort to enhance the reliability of the extracted data.

## Additional file

Additional file 1: Populated PRISMA-P checklist. (DOC 75 kb)

## Abbreviations

AT: Angeline Traynor
BMG: Brian E. McGuire
EM: Eimear Morrissey
EMBASE: Excerpta Medica database
GRADE: Grades of Recommendation Assessment, Development and Evaluation
IASP: International Association for the Study of Pain
JE: Jonathan Egan
JIA: Juvenile idiopathic arthritis
MEDLINE: Medical Literature Analysis and Retrieval System Online Abbreviations
MeSH: Medical Subject Headings
mRCT: metaRegister of Controlled Trials
OR: Odds ratio
PRISMA: Preferred Reporting Items for Systematic Reviews and Meta-Analyses
PRISMA-P: Preferred Reporting Items for Systematic Reviews and Meta-Analyses protocols 2015 statement
RCT: Randomised controlled trials
WHO ICTRP: World Health Organization International Clinical Trials Registry Platform
